# Nasal Mucosa Derived-Mesenchymal Stem Cells from Mice Reduce Inflammation via Modulating Immune Responses

**DOI:** 10.1371/journal.pone.0118849

**Published:** 2015-03-04

**Authors:** Chen Yang, Jing Li, Hai Lin, Keqing Zhao, Chunquan Zheng

**Affiliations:** 1 Department of Otolaryngology-Head and Neck Surgery, Eye Ear Nose and Throat Hospital, Fudan University, Shanghai, China; 2 Department of Otolaryngology, Ruijin Hospital, School of medicine, Shanghai Jiao Tong University, Shanghai, China; Université Libre de Bruxelles, BELGIUM

## Abstract

Mesenchymal stem cells (MSCs) have arisen the attention to be a new attractive therapeutic tool treating autoimmune diseases such as allergic rhinitis (AR). AR is a chronic reversible allergic inflammation caused by the excessive activation of T-helper 2 (Th2) cells. Recently, MSCs have been proposed as a new therapy of AR as it can suppress some cytokines to control allogeneic Th2 response and functions. However, how MSCs function to reduce inflammation remains unclear. In this study, we aimed to investigate the role of ectomesenchymal stem cells (ECTO-MSCs) derived from nasal mucosa in eosinophilic inflammation and how it affects some immunoglobulins and cytokines. We used ovalbumin (OVA) as a sensitizer to induce nasal inflammation in mice by both injection and inhalation. In order to obtain deeper insights into the influences of ECTO-MSCs on nasal inflammation, the migration of ECTO-MSCs was assessed, the numbers of eosinophils and sneezing were counted, and several immunoglobulins and cytokines were measured. Here we show the ECTO-MSCs are able to migrate to inflammation site via tail vein injection. Eosinophils and sneezing were suppressed by ECTO-MSCs. Interestingly, IgE, interleukin (IL)-4, IL-5 and IL-10 secreted by Th-2 cells were down-regulated by ECTO-MSCs whereas IgG_2_ and IFN-γ were up-regulated. In conclusion, we have observed that ECTO-MSCs are associated with enhanced Th-1 immune response to nasal inflammation and reduced Th-2 immune response. Given the contributions of Th-2 cells to AR, the injection of ECTO-MSCs can be a promising therapy of AR through balancing immune response.

## Introduction

Mesenchymal stem cells (MSCs), also referred to as bone marrow stromal stem cells have been defined as a group of adult primitive progenitor cells that can be easily isolated from several tissues such as bone marrow, adipose tissue and menses blood [1, 2]. These cells are capable of self-renewing and multilineage differentiation to generate osteoblasts, adipocytes, myotubes, tenocytes, neural cells and chondrocytes[3]. The pluripotency of MSC make it an attractive therapeutic tool such as treating autoimmune diseases.

Allergic rhinitis (AR) is a chronic reversible allergic condition inducing rhinorrhoea, nasal obstruction, nasal itching and sneezing [4]. AR is characterized by eosinophilic dependent inflammation and T-helper 2 (Th2) excessive activation [5]. Evidence has shown that the Th2 cytokines such as interleukin (IL)-4, IL-5, IL-13 down-regulated by T cells were elevated in AR patients [6]. The symptoms of AR can be reduced by treating with usual pharmacotherapy such as antihistamines and topical nasal corticosteroids whereas immunotherapy is employed if patients are resistant to the usual pharmacotherapy [7]. Allergen immunotherapy involves regular injection of incremental doses of allergen vaccines to accustom suffers to allergens, which is the only treatment that can potentially modifies the process of the disease [8]. However, the mechanism of immunotherapy remains controversial.

Recently, MSCs have been proposed as a new therapy of AR as they are able to suppress the release of cytokines to control allogeneic T-cell response and function as a profound immunomodulator [5]. MSCs can modulate immune systems by affecting several effector functions and also can promote the survival of damaged cells by migrating to injured tissues and inhibiting the releases of proinflammatory cytokines [9]. Researchers have postulated that MSCs play a potential role in modulating allogeneic immune cell responses based on the clinical responses of treating graft-versus-host disease[10–12]. It was also documented that the immunomodulatory effects of MSCs protected against kidney damage by migrating to injured kidney and suppressing inflammation [13]. Therefore, researchers have begun investigating the effects of MSCs on AR. It was demonstrated that MSCs reduced allergen-driven pathology of allergic airway inflammation by decreasing cytokines like IL-4 but increasing of IL-10 [13]. However, it involves multiple regulatory of T cells dependent and independent mechanisms of therapeutic action.

Not much research has investigated the immunomodulatory effects of MSCs obtained from nasal mucosa. In this study, we addressed the immunomodulatory effects of nasal mucosa MSCs on AR, providing a basis of further clinical applications of MSCs on treating allergic diseases.

## Materials and Methods

### Animals

The care and use of animals in this study followed the guidelines and protocol approved by the Institutional Animal Care and Use Committee (IACUC) of Ruijin Hospital. The IACUC committee members at Ruijin Hospital appoved this study. All efforts were made to minimize the number of animals used and their suffering. Mice were kept in a temperature (21±2°C) and humidity (55±10%) controlled room on a 12:12 light dark cycle (light 7AM–7PM). Mice had *ad libitum* access to water and food. When indicated, mice were maintained for 8 weeks and sacrificed. After the experiments, the animals were killed by CO_2_ inhalation followed by decapitation.

### Isolation and culture of MSCs

To isolate ectomesenchymal stem cells (ECTO-MSCs), mice with bodyweight between 250g and 300g were employed. 0.35% pentobarbital sodium (35 mg/kg) was intraperitoneally injected to anesthetize mice. Facial disinfection was implemented and nasal mucosa was obtained under sterile environment by resecting 1/3 of mucosa from nasal septum exposed by extending incision of nasal skin and bone below nares to inner cavity. Nasal mucosa was carefully removed from the nasal septum mucosa in PBS and then washed extensively with DMEM/F12 (v/v = 1:1) (Gibco) containing 100 u/ml penicillin and 100 g/ml streptomycin. Nasal mucosa was digested with 0.075% collagenase type I (Sigma-Aldrich) at 37°C for 30 minutes. Enzyme activity was neutralized with modified Eagle’s medium (DMEM) containing 10% fetal bovine serum (FBS) and the sample was centrifuged at 1200 g for 10 minutes to obtain a pellet. The cells were incubated overnight at 37°C in 5% CO_2_ in control medium (DMEM, 10% FBS, 100 U /ml penicillin, 100 g/ml streptomycin) at a concentration of 1×10^6^ cells/ml in Corning flask. After 3 days, cells were trypsinized (0.05% trypsin-EDTA, Sigma-Aldrich), resuspended in DMEM containing 10% FBS. Culture medium was changed every 3 days. The passage 4 was used in these experiments.

### Multilineage differentiation of MSCs

MSCs were analyzed for their multipotency to differentiate toward the myogenic adipogenic and osteogenic lineages by culturing in various conditioned media. Myogenic differentiation was induced after MSCs became confluence by culturing MSCs at low density in normal growth medium and after 24 hours, the medium was changed to myogenic induction medium consisting of low glucose DMEM supplemented with 10% FBS, 10 μM 5-azacytidine, and 10 ng/ml basic FGF. The myogenic induction medium was changed to normal growth medium supplemented with 10 ng/ml basic FGF after 24 hours. Culture medium was replaced every 3 days. After 2 weeks induction, cells were fixed, stained for skeletal heavy chain myosin, and their morphological characteristics, especially the presence of myotube-like organization were also identified.

Adipogenic differentiation was induced after MSCs became confluence by culturing MSCs for 2 weeks in adipogenic medium (1 μM dexamethasone, 100 μg/ml 3-isobutyl-1-methylxanthine, 5 μg/ml insulin, 60 μM indomethacin, and 10% FBS in high-glucose DMEM) and was stained with Oil Red O, an indicator of intracellular lipid accumulation. The MSCs were fixed at room temperature in 70% ethanol for 15 minutes and incubated in 2% Oil Red O reagent for 1 hour at room temperature. 70% ethanol was employed to wash excess stain, followed by several changes of distilled water.

Osteogenic differentiation was induced after MSCs became confluence by culturing MSCs for 2 weeks in osteogenic medium (0.1 mM dexamethasone, 10 μM β-glycerophosphate, 50 μg/ml ascorbic acid, and 10% FBS in low-glucose DMEM) and assessed for extracellular matrix calcification by alizarin red S staining. MSCs were fixed with 70% ethanol and washed with distilled water. The cells were incubated in 2% alizarin red solution for 15 minutes at room temperature and washed for 3 times with distilled water

### Induction of nasal mucosa inflammation

Mice were sensitized using ovalbumin (OVA) (Sigma) and aluminum hydroxide (Alum) (Sigma). Mice were sensitized via tail vein injection of 20 μg OVA emulsified in 1mg Alum in a total volume of 200 μl PBS, and boosted with the identical antigen after 14 days. 4 days later, purified ECTO-MSCs derived from nasal mucosa were injected via the tail vein for 3days. On day 21, 22 and 23 after the initial sensitization, these mice were challenged for 30 minutes with an aerosol of 5% OVA in PBS in a plexiglass chamber through an ultrasonic nebulizer (NE-U12, Omron, Japan).

Mice were divided into four groups, with eight mice in each group. In control group, mice were sensitized, treated, and challenged with PBS. In ECTO-MSC group, mice were sensitized and challenged with PBS, but ECTO-MSCs were injected via the tail vein as described above. In OVA group mice were sensitized with OVA and Alum and challenged with OVA, but instead of injection of ECTO-MSCs, PBS was injected. In OVA+ESC group, mice were sensitized with OVA plus Alum, injected ECTO-MSCs, and challenged with OVA.

### Immunofluorescence staining

Mice were divided into three groups. In control group, only PBS was used to treat on mice. Saline group and MSC group were injected by saline and MSCs respectively before the first challenge of OVA at 21^st^ Day. Nasal mucosa sections removed from mice were washed in cold PBS and sliced into 7 μm by cryostat microtome (Leica) and fixed by 4% paraformaldehyde for 24 hours at room temperature. Diluted Nestin and DAPI were respectively utilized to stain each specimen, which was kept in dark for 30 minutes at room temperature, and were washed by PBS. Fluorescence microscope was used to observe the results.

### MSCs migration assessment

MSCs were purified and labeled with 2 μM Cell Tracker CM-Dil (Molecular Probes Inc., Eugene) at 37°C for 5 minutes, and then incubated at 4°C for 15 minutes. MSCs were washed with PBS and suspended in PBS at a concentration of 2×10^7^ cells/ml. 0.1 ml of purified stem cells was injected with a 26-gauge needle via the mouse tail vein on day 18, 19, and 20. 24 hours after the final inhalation of OVA, mice were killed. Nasal mucosa was removed, fixed, stained with DAPI and then observed by fluorescence microscopy.

### Measurement of inflammation and nasal allergic symptoms

Nasal mucosa was removed and washed by PBS, and then immersed in 10% neutral buffered formalin and fixed in the 4% paraformaldehyde for 24 hours. After that, nasal mucosa was embedded in Technovit 7100 resin (Heraeus Kulzer, Wehrheim). Inflammation severity was determined by staining with hematoxylin and eosin, and observed by light microscopy with a high-power field. The infiltration effect and numbers of eosinophils were compared between four mice groups. Tissue sections were randomly selected when counting. The absolute numbers of eosinophils were counted as mean standard error of the mean. Nasal allergic symptoms were assessed by counting the number of sneezing per hour and observers were not told the group of mice.

### Detection of immunoglobulin levels

24 hours after the final inhalation of OVA, blood samples were collected and tested by enzyme-linked immunosorbent assay (ELISA) using an ELISA kit (Abcam, UK) and based on the instruction of the manufacturer. 96 wells microtiter plates were coated with isotype-specific goat anti-mouse mAbs (IgE, IgG_1_, IgG_2a_) diluted in PBS and 2% bovine serum albumin (BSA) and incubated overnight at 4°C. Plates were extensively washed and serially diluted serum samples (100 μl/ well) were added in duplicate and incubated at 37°C for 2 hours in a humidified atmosphere. After washing with 0.1% Tween 20 in PBS (PBS-T), horseradish peroxidase-conjugated anti-mouse IgE (Pharmingen, Hamburg, Germany) (1:4,000), IgG_1_ (1:5,000), or IgG_2a_ (1:5,000) was added to each well and incubated at room temperature for 1 hour. The plate was washed by PBS-T and developed with 100 μl tetramethyl benzidine (Sigma-Aldrich). Absorbance (450 nm) was measured with an ELISA plate reader (Molecular Devices Corporation, Sunnyvale, CA).

### Expression of cytokines in the spleen

The spleen was removed 24 hours after the last OVA challenge, and collected in a tissue culture petri dish containing 3 ml of culture medium. 5-ml syringe was applied to crush the tissue. Then, the crushed tissue was treated with lysis buffer containing ammonium chloride and potassium bicarbonate to deplete erythrocytes. Single-cell suspensions of splenocytes (2×10^5^ cells/well in 96-well culture plates) were cultured in RPMI 1640 medium supplemented with 10% FBS, 2 mM glutamine, 1 mM sodium pyruvate, and antibiotics with OVA (100 ng/ml). Interleukin (IL)-4, IL-5, IL-10, and interferon (IFN)-γ levels were measured after 2 days culture by ELISA using an ELISA kit (Abcam, UK) followed the instruction of manufacturer.

### Statistical analysis

Data are presented as mean standard error of the mean. Comparisons between groups were made by the Kruskall-Wallis test followed by Dun’s test using the SPSS software package version 12.0 (SPSS Inc., Chicago, IL) and p<0.05 was considered as statistical significant.

## Results and Discussion

### Identification of ECTO-MSCs in nasal mucosa

**Fig 1:**
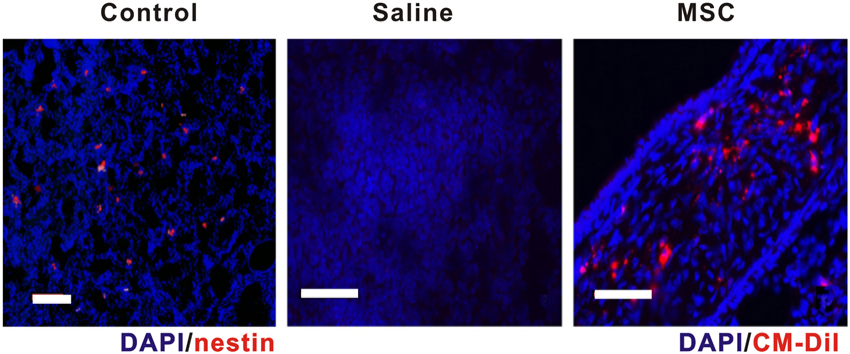
Immunofluorescence of injected ECTO-MSCs in nasal mucosa. MSCs were labeled with DAPI and nestin (A), indicating endogenous MSCs exist in nasal mucosa of mouse. CM-Dil staining as cell tracker shows the migration of injected MSCs to nasal mucosa via tail vein injection (C) compared to saline control (B) (scale bar = 200 μm).

To determine whether endogenous ECTO-MSCs exist in the nasal mucosa in mouse, sections from nasal mucosa were stained with DAPI for nucleus and nestin utilized as a marker for stem cells. Fluorescence microscopy was employed to visualize the sections. It was already reported that a niche of ECTO-MSCs displaying the propensity to differentiate to neural cells and osseous cells, suggesting the existence of MSCs in the mucosa of nasal cavity [14]. Nestin positive cells were found in nasal mucosa section, shown as red in Fig. 1A, demonstrating that MSCs are widespread in the nasal mucosa in normal mice. To further explore whether the exogenous MSCs migrate to the nasal mucosa, saline or purified ECTO-MSCs were injected before the first allergen challenge of two groups of AR mice. ECTO-MSCs were labeled with Cell Tracker CM-Dil. To compare the results between saline and MSCs injection groups, migration of MSCs to nasal mucosa was observed as labeled MSCs were found accumulated in nasal mucosa of mice treated with MSCs injection (Fig. 1C), while nearly no exogenous MSC could be found in saline group (Fig. 1B). It demonstrates that MSCs were able to migrate to nasal mucosa of mice via the tail vein injection, which provide the basis of further investigation on the effects of MSCs on inflammatory responses. Previous study also proved the migration of MSCs derived from adipose tissue to the inflammation sites of nasal mucosa in allergic rhinitis mice [15]. In another word, the immune system may recruit MSCs to adjust the inflammatory environment.

### Growth and differentiation characterization of nasal mucosa-derived MSCs

**Fig 2:**
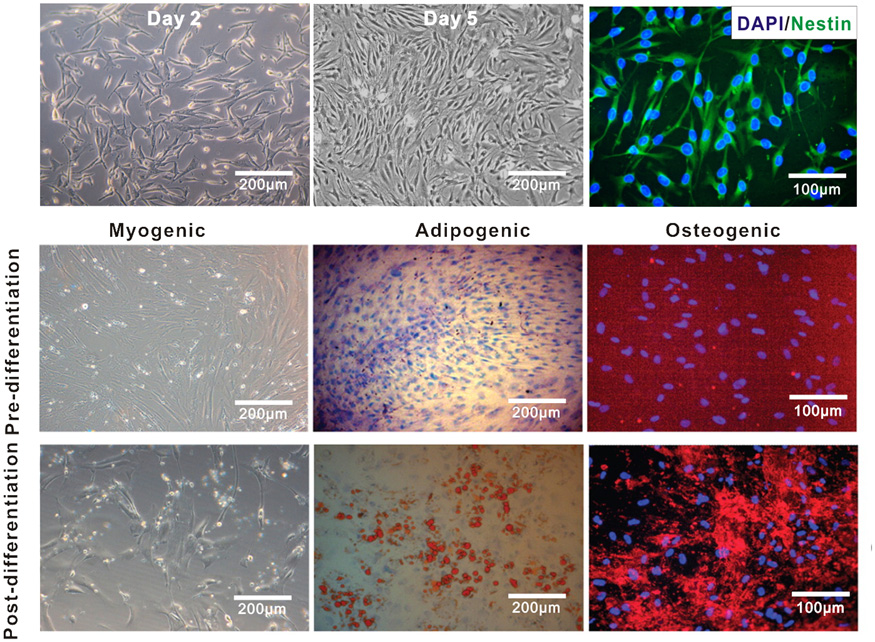
The growth of ECTO-MSCs and differentiation of ECTO-MSCs into myocytes, adipocytes and osteocytes. Phase contrast images of nm-MSCs in day2 (A) and day 5 (B). ECTO-MSCs (C) were stained with DAPI and Nestin. Myogenic differentiation was shown by phase contrast images (A and G). Adipogenic differentiation was indicated by accumulation of neutral lipid vacuoles that stained with Oil Red O (E and H). Osteogenic differentiation was indicated by nuclei stained with Hoechst and calcium deposits visualized with Alizarin Red (F and I).

To know whether ECTO-MSCs can be cultured in vitro, MSCs from nasal mucosa of mice were isolated and cultured. We observed a dramatically increasing of cell density from day 2 to day 5 (Fig. 2A and B). Most of cells adhered to the surface of flask after 3 days and the growth tended to be confluence at day 5. DAPI and nestin staining (Fig. 2C) result identified the cells as stem cells. These results demonstrate that the ECTO-MSCs are able to proliferate to generate identical copies of themselves, which is one of the defining characteristics of stem cells [16].

The multilineage differentiation potential of ECTO-MSCs was displayed by culturing cells with respective treatments as described in differentiation methods. Myogenic differentiation was apparent after 2 weeks as the reduction in size and acquisition of myoblast as well as the formation of myotube morphologies (Fig. 2D and G). By contrast, ECTO-MSCs obtained from same colonies differentiated into adipocytes after 2 weeks with adipogenic medium treatment, which is shown by the accumulation of Oil Red O staining lipid rich vacuoles (Fig. 2E and H). These ECTO-MSCs also formed aggregates and calcium deposits (Fig. 2F and I), indicating the differentiation ability of these MSCs to osteogenic MSCs. The results suggest that clonally-derived ECTO-MSCs were multipotent and responsive to differentiation stimuli. This property has been identified as another key characteristic of MSCs [17].

### Allergic analysis of nasal mucosa

**Fig 3:**
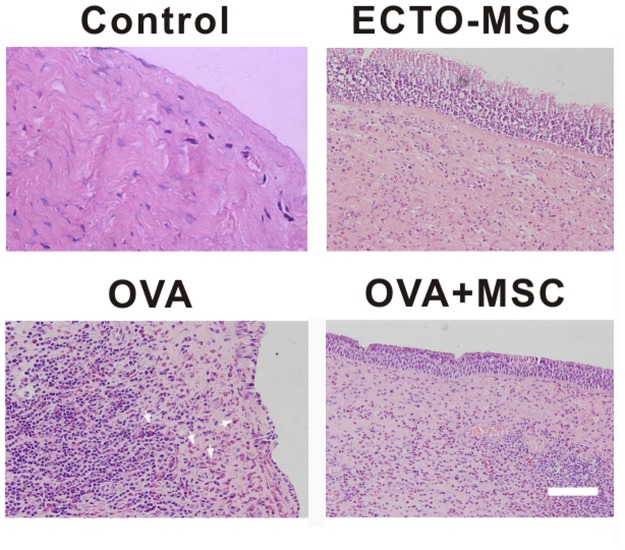
Histological analysis of nasal mucosa. Control (A) and ECTO-MSC (B) groups had neglectable inflammatory eosinophils infiltration into the nasal mucosa. The extravasation of eosinophils was shown in mice sensitized by OVA (C). After MSCs injection, eosinophils were impressively reduced (D). Nasal mucosa sections were stained with hematoxylin and eosin with 200× magnification. Scale bar = 500 μm.

Eosinophil accumulation at the inflammation sites has been proposed to be correlated with allergic diseases [18]. To investigate the influence of MSCs on the inflammation of nasal mucosa, histological examination and hematoxyliln and eosin staining were performed, which shows the changes of inflammatory cells such as eosinophils. In control group (Fig. 3A), no eosinophils were observed, indicating there was no inflammation. There was a slightly inflammatory effect after ECTO-MCSs injection in the nasal mucosa (Fig. 3B). At the second day after mice were sensitized by OVA, nasal mucosa of mice were removed and underwent histological examination for further comparison between OVA group and OVA+MSC group. The injection of MSCs via tail vein significantly reduced infiltration of eosinophils (Fig. 3C and D). Eosinophilic infiltration was documented conspicuously mounting by allergic airway inflammation [19]. As an indicator of clinical severity of airway inflammation, the reduction of eosinophilic infiltration demonstrates the inhibition of MSCs on eosinophilic inflammation.

**Fig 4:**
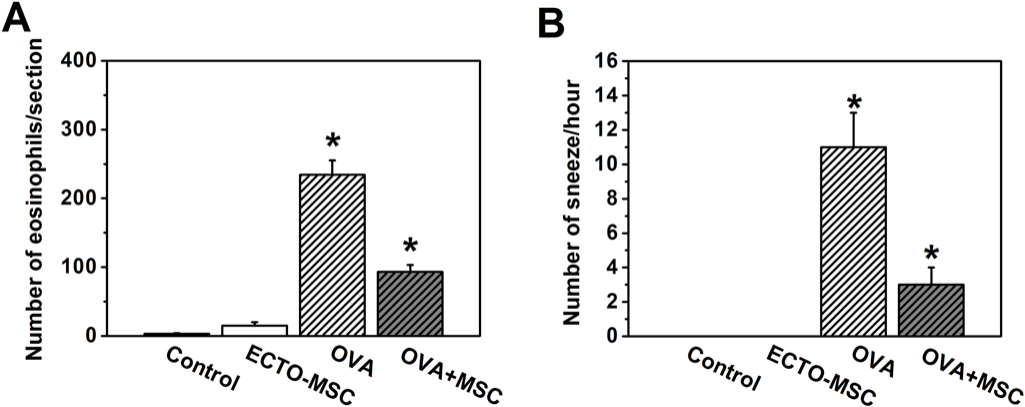
Numbers of eosinophils in each section of nasal mucosa and sneeze numbers per hour. (A) Eosinophils in mice stimulated by OVA expressed at a high level, and the expression was significantly reduced to the injection of purified ECTO-MSCs. Nearly no eosinophilic inflammation was observed in control and ECTO-MSC groups. (B) Sneeze numbers in OVA+MSCs groups were significantly lower in OVA+MSC group than those in the OVA group. No sneeze was observed in control and ECTO-MSC groups. Data are expressed as the mean standard error of the mean (n = 8 in each group). Results are representative of three independent experiments. *, p<0.05 for significant differences.

In order to assess the anti-inflammatory effects of MSCs on allergic symptoms, we also compared the numbers of eosinophils (Fig. 4A) and counted the numbers of sneezing per hour (Fig. 4B) in control, ECTO-MSC, OVA and OVA+MSC groups. The results are complementary to histological results. Nearly no inflammatory cell infiltration was shown in nasal mucosa in control group and ECTO-MSC group, shown as the low number of eosinophils in these two groups. In comparison to OVA group, the number of eosinophils significantly decreased in the presence of MSCs, accounting for the inhibition of inflammation by MSCs. On the other hand, control group and ECTO-MSC group were totally devoid of any sneezing. The sneezing frequency in OVA group was significantly higher than control group, which was dropped by tail vein injection of MSCs, suggesting the improvement of allergic symptoms by MSCs.

### MSCs alter the secretion of immunoglobulin

**Fig 5:**
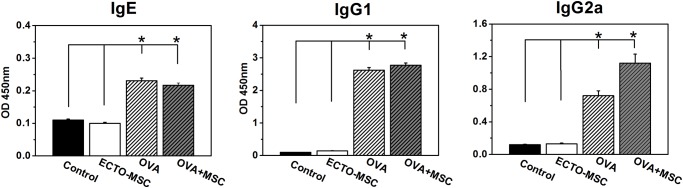
Analysis of serum antigen-specific-antibody responses. The levels of IgE, IgG_1_ and IgG_2a_ in the OVA group are significantly higher than those in the control group. The injection of MSC via tail vein significantly reduced IgE level and slightly improved IgG_1_ level. In contrast, IgG_2a_ production was dramatically improved in OVA+MSC group compared to OVA group. Data are expressed as the mean ± standard error of the mean. (n = 7 in each group). Results are representative of three independent experiments. *, p<0.05 for significant differences.

Since immunoglobulins play important roles in mediating inflammatory reactions and hypersensitivity, we investigated the expressions of several main immunoglobulin antibodies that have been implicated in B-cell immune responses controlled by cytokines from Th cells. IgE, IgG_1_ and IgG_2a_ levels were determined. As shown in Fig. 5, the level of IgE, a Th2 dependent antibody (Anti-interleukin-10 antibody) was significantly higher in OVA groups than that in control group (p<0.05). Interestingly, the induction of MSCs had a statistically significant tendency to reduce the secretion of IgE, indicating MSCs may down-regulate Th2 immune responses. No impressive difference of IgG1 levels was observed between OVA and OVA+MSC groups. In contrast, IgG_2a_ level was significantly higher in OVA+MSC group than OVA group (p<0.05). This reveals the Th1 immune response may be priming by the mounting of IgG_2a_ production [20]. In another words, the maintenance of IgG_1_ and increase of IgG_2a_ suggests the shift of Th2 to Th1 immune response. Studies on the balance Th2 and Th1 documented that atopic individuals possess skewed cytokine expression profiles towards Th2 compared to non-atopic individuals [21]. This shift may be responsible for the reduction of allergic inflammation by MSCs induction.

**Fig 6:**
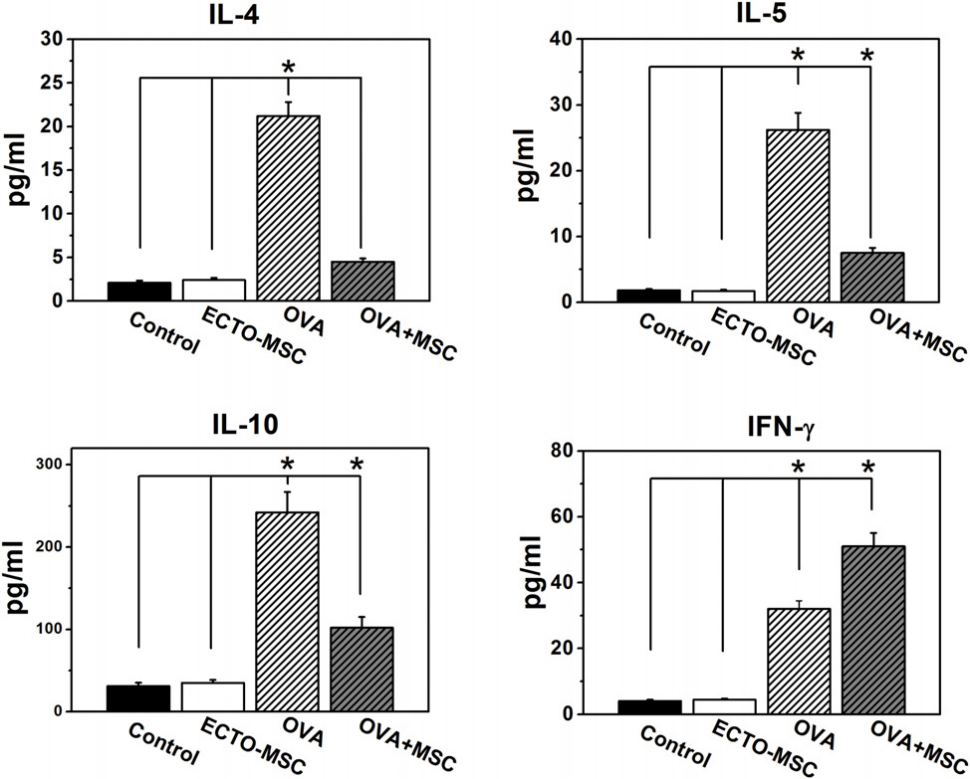
Cytokine changes in supernatants the splenocytes. IL-4, IL-5, IL-10 and IFN-γ were all significantly elevated by OVA stimulation in the OVA group compared to the control group. The injection of MSC in the OVA+MSC group conspicuously decreased IL-4, IL-5 and IL-10 levels but increased IFN-γ level. Data are expressed as the mean ± standard error of the mean. (n = 7 in each group). Results are representative of three independent experiments. *, p<0.05 for significant differences.

Given the reciprocally regulatory characteristic between Th1- and Th2-associated cytokines, we also test the levels of several interleukins and interferon in spleen. As shown in Fig. 6, the levels of IL-4, IL-5 and IL-10 in the supernatants of OVA sensitized splenocytes were impressively elevated compared to control groups. It was reported that IL-4 and IL-5 inhibit the functions of Th1 immune response[22]. IL-5 was stated as an essential modulator of the mobilization and accumulation of eosinophils at the inflammation sites by interacting with the chemokine eotaxin [18]. IL-10 also plays a crucial role in down-regulate the activities of Th1 [23]. All of these interleukins are secreted by Th2 cells [24]. OVA+ MSC group shows lower levels of IL-4, IL-5 and IL-10, therefore demonstrating the inhibition of Th1-associated function from Th2 immune response was delayed. The allergic inflammation controlled by these interleukins such as IL-4 and IL-5 mediating eosinophilic inflammation can be also reduced by MSCs. IL-4 and IL-5 secreted from Th2 cytokines were shown at high level in AR mice, which demonstrate the predominant of Th2 immune response in atopic individuals. Therefore, the reduction of IL-4 and IL-5 also support the proposal that shift from Th2 to Th1 occurs after MSC injection.

In contrast, IFN-γ was increased in the presence of OVA and MSC compared to OVA group. As a cytokine secreted by Th1 cells, which triggers the production of macrophages and also inhibits Th2 cell proliferation [24], the increase of IFN-γ in OVA+MSC group provide another evidence of the ability of MSC on shifting Th2 to Th1 immune response to allergens. However, the balance between Th1 and Th2 is crucial to avoid AR. Since the excessive activation of Th2 was identified to be a leading cause of AR, the shift from Th2 to Th1 caused by MSCs injection may be a feasible way to control AR. Further studies are required to gain deeper insights into the influence of MSCs on modulating Th1 and Th2 response in nasal allergic inflammation.

In conclusion, this study investigated the migration of ECTO-MSCs to nasal mucosa via tail vein injection in OVA sensitized mice. MSC was proved to be a modulator of Th1 and Th2 immune response balance, by the rising of IgG_2a_ and IFN-γ but dropping of IgE, IgG_1_, IL-4, IL-5 and IL-10. Our study strongly proves the existence of endogenous MSCs in nasal mucosa of mice and provides the possibility of employing MSCs to treat allergic diseases. However, further studies are still needed to explore the mechanisms underlying the immune modulatory capabilities of MSCs.
